# Massive hemobilia caused by rupture of gastroduodenal artery pseudoaneurysm, a delayed complication of laparoscopic cholecystectomy: a case report

**DOI:** 10.1186/s13256-021-02915-1

**Published:** 2021-07-02

**Authors:** Kurniawan Kurniawan, I Dewa Nyoman Wibawa, Gde Somayana, I Ketut Mariadi, I Made Mulyawan

**Affiliations:** 1 Department of Internal Medicine, Faculty of Medicine, Udayana University/Sanglah General Hospital, Denpasar, Bali Indonesia; 2 Gastroentero-Hepatology Division, Department of Internal Medicine, Udayana University/Sanglah General Hospital, Denpasar, Bali Indonesia; 3 Digestive Surgery Division, Department of General Surgery, Faculty of Medicine, Udayana University/Sanglah General Hospital, Denpasar, Bali Indonesia

**Keywords:** Hemobilia, Pseudoaneurysm, Gastroduodenal artery pseudoaneurysm, Laparoscopic cholecystectomy

## Abstract

**Background:**

Hemobilia is a rare cause of upper gastrointestinal bleeding that originates from the biliary tract. It is infrequently considered in diagnosis, especially in the absence of abdominal trauma or history of hepatopancreatobiliary procedure, such as cholecystectomy, which can cause arterial pseudoaneurysm. Prompt diagnosis is crucial because its management strategy is distinct from other types of upper gastrointestinal bleeding. Here, we present a case of massive hemobilia caused by the rupture of a gastroduodenal artery pseudoaneurysm in a patient with a history of laparoscopic cholecystectomy 3 years prior to presentation.

**Case presentation:**

A 44-year-old Indonesian female presented to the emergency department with complaint of hematemesis and melena accompanied by abdominal pain and icterus. History of an abdominal trauma was denied. However, she reported having undergone a laparoscopic cholecystectomy 3 years prior to presentation. On physical examination, we found anemic conjunctiva and icteric sclera. Nonvariceal bleeding was suspected, but esophagogastroduodenoscopy showed a blood clot at the ampulla of Vater. Angiography showed contrast extravasation from a gastroduodenal artery pseudoaneurysm. The patient underwent pseudoaneurysm ligation and excision surgery to stop the bleeding. After surgery, the patient’s vital signs were stable, and there was no sign of rebleeding.

**Conclusion:**

Gastroduodenal artery pseudoaneurysm is a rare complication of laparoscopic cholecystectomy. The prolonged time interval, as compared with other postcholecystectomy hemobilia cases, resulted in hemobilia not being considered as an etiology of the gastrointestinal bleeding at presentation. Hemobilia should be considered as a possible etiology of gastrointestinal bleeding in patients with history of cholecystectomy, regardless of the time interval between the invasive procedure and onset of bleeding.

## Background

Hemobilia was first described as a cause of upper gastrointestinal bleeding (UGIB) in 1654, by Francis Glisson. Hemobilia is defined as bleeding of the biliary tract derived from the biliary system itself, the gall bladder, or the orifice of the ampulla of Vater. Its clinical presentation is known as the Quincke’s triad, including upper abdominal pain, icterus, and UGIB [1].

Incidence of hemobilia has increased along with the increased practice of hepatopancreatobiliary invasive procedures [2]. Cholecystectomy is one of the etiologies of hemobilia caused by arterial pseudoaneurysm. Although rare, hemobilia itself has a high mortality rate and is often unrecognized during the diagnosis establishment, especially in cases without history of abdominal trauma or hepatopancreatobiliary procedure [3]. Moreover, early diagnosis of hemobilia is crucial because its management strategy is distinct from the other etiology of UGIB [1].

To increase awareness towards this condition, we present a case of massive hemobilia caused by rupture of a gastroduodenal artery pseudoaneurysm in a patient with history of laparoscopic cholecystectomy (LC) 3 years prior to presentation.

## Case presentation

A 44-year-old Indonesian female patient presented to the Emergency Department of Sanglah General Hospital (Bali, Indonesia) with the chief complaint of blackish stool that had occurred 2 hours prior to admission and was accompanied by coffee ground vomitus and epigastric pain. Two weeks prior to admission, the patient had been admitted to the district hospital with complaint of blackish stool and received a blood transfusion. The blackish stool recurred 1 week later.

The patient denied a history of abdominal trauma but reported a history of elective LC due to cholelithiasis 3 years prior to admission and was hospitalized for 5 days. History of abdominal pain or gastrointestinal bleeding afterwards was denied. History of alcoholism, hepatitis, or chronic liver diseases was denied.

The patient’s vitals were within normal range; blood pressure was 110/70 mmHg, heart rate was 84 beats per minute, respiratory rate was 16 breaths per minute, and axillary temperature was 36.6°C. Pallor of the conjunctiva indicated anemia, and scleral icterus suggested slight jaundice. The patient expressed pain on palpation at the epigastric and right hypochondriac region.

Blood analysis revealed low hemoglobin 9.88 g/dL (normal range: 12–15.5 g/dL) but normal platelet and white blood cell count. Liver function markers were abnormal, with aspartate transaminase of 354.9 U/L (11–33 U/L), alanine transaminase of 321.3 U/L (11–50 U/L), total bilirubin of 3.52 mg/dL (0.3–1.3 mg/dL), direct bilirubin of 2.95 mg/dL (0.0–0.3 mg/dL), alkaline phosphatase of 233 U/L (53–128 U/L), and gamma-glutamyl transferase of 301 U/L (70–140 U/L). Amylase, lipase, prothrombin, and partial thromboplastin times were normal.

Results from abdominal ultrasonography were unremarkable; there were no signs of biliary obstruction or intraabdominal free fluid. Nonvariceal UGIB caused by peptic ulcer was suspected, and appropriate conservative treatment with lansoprazole bolus 60 mg intravenously followed by continuous drip 6 mg/hour intravenously was initiated, with esophagogastroduodenoscopy (EGD) ordered for further evaluation.

**Fig. 1:**
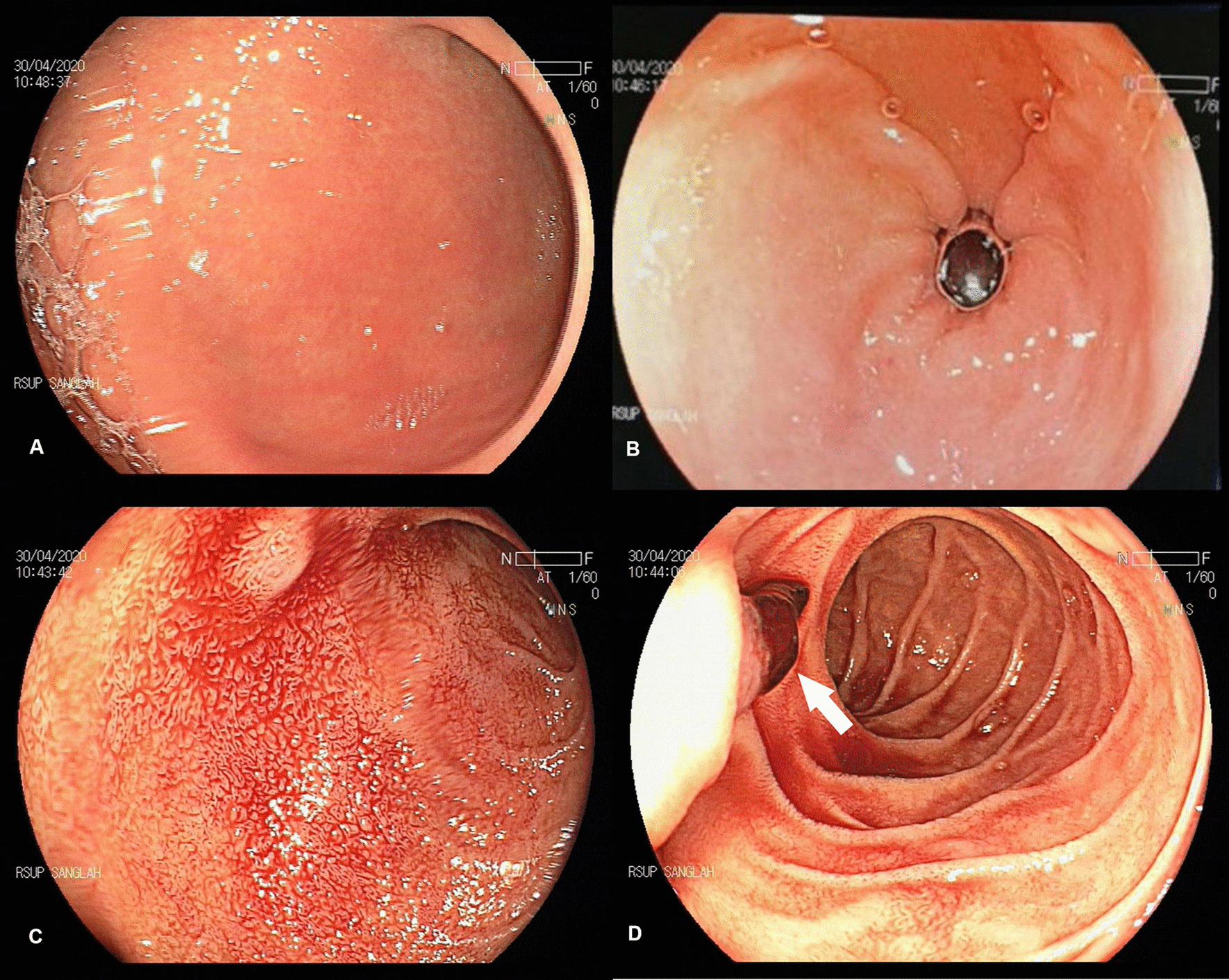
Esophagogastroduodenoscopy result: no source of bleeding apparent after cleansing of the gastric region (**A**, **B**). Duodenal bulb covered by blood (**C**). Blood clot found at the ampulla of Vater (white arrow) (**D**)

The EGD revealed blood covering the gastric tissues up to the second part of the duodenum. After cleansing, the source of bleeding remained inapparent. However, a blood clot was found at the ampulla of Vater (Fig. 1). The diagnosis of hemobilia was made, and computed tomography (CT) scan was planned

**Fig. 2:**
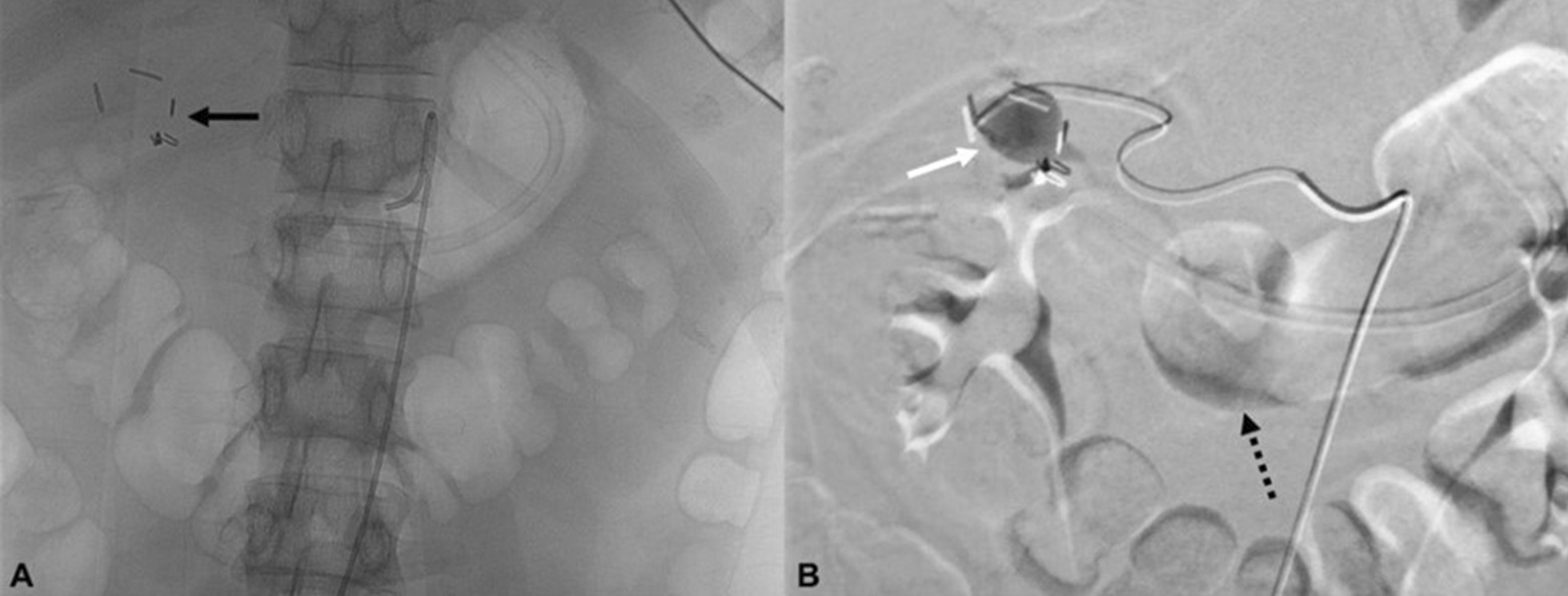
Angiography examination showed gastroduodenal artery pseudoaneurysm: with no contrast administration, a surgical clip was visualized (black arrow) (**A**); after contrast administration, a pseudoaneurysm was visualized in the surgical clip area (white arrow), along with contrast extravasation to the duodenum that refluxed to the stomach (dotted arrow) (**B**)

While waiting for the abdominal CT scan, the patient experienced massive hematemesis and hematochezia. Emergent angiography was performed to determine the source of bleeding and address the patient’s hemodynamic instability. The imaging showed contrast extravasation from a gastroduodenal artery pseudoaneurysm distal to the common bile duct, with flow to the duodenum (Fig. 2). The patient was diagnosed with hemobilia due to ruptured gastroduodenal artery pseudoaneurysm.

**Fig. 3:**
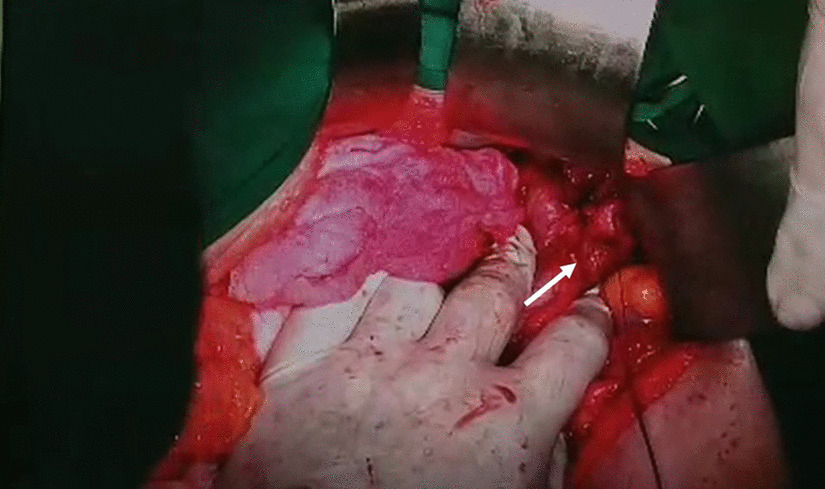
Ligation of gastroduodenal artery pseudoaneurysm. White arrow showed a ligated gastroduodenal artery pseudoaneurysm

The patient was treated with fluid resuscitation and packed red cell transfusion. Although transcatheter arterial embolization would have been the preferred procedure to stop the bleeding, it was unavailable in our center; therefore, laparotomy surgery was performed. The laparotomy confirmed the bleeding from gastroduodenal artery pseudoaneurysm, and ligation and excision of the pseudoaneurysm were performed (Fig. 3). After surgery, the patient’s vital signs were stable, and no recurrent bleeding episode occurred. Unfortunately, the patient developed ventilator-associated pneumonia and passed away.

## Discussion

In this case report, we present a patient with characteristics concordant with the Quincke’s triad [4]. Hemobilia is a rare etiology of UGIB and often missed in the early diagnosis establishment, especially when there is no history of trauma or invasive hepatopancreatobiliary procedure [1]. Historically, trauma has been the most frequent cause of hemobilia, but Green *et al.* [5] reported on a change in the etiological profile; specifically, the overall increase in patients undergoing invasive hepatopancreatobiliary procedures had led to an iatrogenic etiology becoming the most common cause of hemobilia, accounting for 65% of cases.

Surgery performed around the biliary tract, such as cholecystectomy, liver transplant, or pancreatoduodenectomy, carries risk of causing concurrent injury to the surrounding blood vessels and biliary tract, which can lead to fistula formation [2, 6]. Furthermore, exposure of bile from a leaking biliary duct into injured blood vessels could impair the healing process and cause pseudoaneurysm [7].

The patient presented herein had denied any history of abdominal trauma. However, she had a history of LC three years prior to admission. In LC, vascular injury can be caused by the mechanical maneuvers during surgical clip placement or cystic duct resection, by erosion of the blood vessels by the clip itself, or by heat (thermal injury) directly from the cauterization or indirectly transferred by the clip [8, 9].

In general, hemobilia occurs within 4 weeks after trauma or invasive procedure, while hemobilia caused by endoscopic retrograde cholangiopancreatography or percutaneous biliary drainage usually occurs immediately or within days [4]. Hemobilia due to pseudoaneurysm rupture may occur several years after the invasive procedure [9], but such cases are rare. The delayed onset could be due to a slowly progressing pseudoaneurysm but could result in a massive bleeding event [10, 11].

Tessier *et al.* [12] reported a mean time of 5.7 months between initial invasive intervention and diagnosis of hepatic artery pseudoaneurysm, with the longest interval being 38 months after the procedure. There are two case reports in the literature of delayed-onset hemobilia that occurred after more than 1 year. The first, by Badillo *et al.* [8], reported on a hemobilia case that occurred 15 months after LC. The second, by Kumar *et al.* [13], reported on a hemobilia case that, similar to our case, occurred 3 years after LC.

Hemobilia should be suspected in UGIB with an unknown source, especially if there is a history of abdominal trauma or hepatopancreatobiliary procedure [2, 4]. Any signs and symptoms of biliary obstruction, both on clinical and laboratory examinations, in UGIB patients with unknown cause should be suspected as hemobilia [4, 6].

Symptoms of hemobilia depend on the source and amount of bleeding, which could be hematemesis, melena, hematochezia, or a combination of these [10, 11]. Postpercutaneous invasive procedure hemobilia is characterized by bleeding from the drainage catheter [4]. Massive hemobilia, such as in our case, may show symptoms of hematochezia, but this complicates diagnosis, risking the misdiagnosis of lower gastrointestinal bleeding [2].

EGD is a common early method applied in the diagnosis of patients with UGIB, especially when there is no suspicion of hemobilia [3, 7]. Blood flowing from or a blood clot at the ampulla of Vater, as detected by EGD, confirms the diagnosis of hemobilia. Furthermore, EGD is also useful for ruling out other sources of bleeding [1]. The success of EGD in detecting hemobilia depends on the duration and severity of the bleeding [6]. Gandhi *et al.* [13] reported that 62% of hemobilia cases that underwent EGD showed bleeding in the second part of the duodenum.

In suspected cases of hemobilia with stable hemodynamics, the diagnostic procedure could be conducted with computed tomography angiography (CTA) without previous EGD [7]. CTA is capable of determining the location of bleeding but unable to determine severity of the bleeding [1, 2]. CTA in patients with hemobilia will show contrast extravasation, blood clot in the biliary system, or vascular malformation [11, 14]. The key advantages of CTA are its noninvasive nature, lower radiation exposure, and provision of a rapid result. The subsequent hepatobiliary vascularization reconstruction can also guide the selection of embolization location, especially in patients with anatomical transformations, for example the post-liver transplant patient [7, 10].

Angiography has become the first choice for diagnosis of hemobilia, and radiological intervention is the preferred treatment modality, especially for patients with unstable hemodynamics [2, 13]. Angiographic technology allows for visualization of the vascular anatomy and localization of bleeding, as marked by contrast extravasation to the biliary system in hemobilia. Moreover, it may also show any arteriobiliary fistula or vascular malformation (aneurysm, pseudoaneurysm, or hemangioma) [15, 16]. One study determined that angiography is remarkably superior to CTA for detecting pseudoaneurysm (100% versus 67%) [10].

The hepatic artery is the most common location of post-LC pseudoaneurysm, followed by the cystic artery [11, 13, 14]. Cases of gastroduodenal artery pseudoaneurysm are rare, accounting for only 1.5% of all reported cases of visceral artery aneurysm; however, the majority of gastroduodenal artery pseudoaneurysms are caused by pancreatitis [17]. To our knowledge, the case described herein is the fourth reported case of hemobilia due to ruptured gastroduodenal artery pseudoaneurysm after cholecystectomy [18–21].

Because hemobilia is a rare etiology of UGIB, it should be suspected in UGIB with unknown source, especially in patients with a history of abdominal trauma or hepatopancreatobiliary procedure, regardless of the time interval between the procedure and the onset of bleeding.

## Conclusion

Gastroduodenal artery pseudoaneurysm is a rare complication of cholecystectomy. To our knowledge, this is the fourth reported case of hemobilia due to ruptured gastroduodenal artery pseudoaneurysm after cholecystectomy. The prolonged time interval between cholecystectomy and bleeding onset (3 years), compared with other cases of postcholecystectomy hemobilia, resulted in hemobilia not being considered as the etiology of UGIB at the patient’s first presentation. Therefore, this case highlights the importance of hemobilia to be considered as a possible etiology of UGIB, especially when there is a history of hepatopancreatobiliary procedure, regardless of the time interval between the invasive procedure and onset of bleeding.

## Data Availability

Not applicable.

## Abbreviations

UGIB: Upper gastrointestinal bleeding
LC: Laparoscopic cholecystectomy
EGD: Esophagogastroduodenoscopy
CT: Computed tomography
CTA: Computed tomography angiography
